# Fly Pollination of Kettle Trap Flowers of *Riocreuxia torulosa* (Ceropegieae-Anisotominae): A Generalized System of Floral Deception

**DOI:** 10.3390/plants10081564

**Published:** 2021-07-29

**Authors:** Annemarie Heiduk, Ulrich Meve, Frank Menzel, Jean-Paul Haenni, Michael von Tschirnhaus, Stefan Dötterl, Steven D. Johnson

**Affiliations:** 1 Centre for Functional Biodiversity, School of Life Sciences, University of KwaZulu-Natal, Private Bag X01, Scottsville, Pietermaritzburg 3209, South Africa; JohnsonSD@ukzn.ac.za; 2 Department of Plant Systematics, University of Bayreuth, Universitätsstrasse 30, 95440 Bayreuth, Germany; ulrich.meve@uni-bayreuth.de; 3 Senckenberg Deutsches Entomologisches Institut, Eberswalder Straße 90, 15374 Müncheberg, Germany; frank.menzel@senckenberg.de; 4 Entomology, Muséum d’Histoire Naturelle Neuchâtel, Rue des Terreaux 14, 2000 Neuchâtel, Switzerland; jean-paul.haenni@unine.ch; 5 Faculty of Biology, University of Bielefeld, Bielefeld, Universitätsstraße 25, 33615 Bielefeld, Germany; m.tschirnhaus@uni-bielefeld.de; 6 Department of Biosciences, Plant Ecology, Paris Lodron University of Salzburg, 5020 Salzburg, Austria; Stefan.Doetterl@sbg.ac.at

**Keywords:** Apocynaceae-Asclepiadoideae, electroantennography, *Ceropegia*, flower scent, fly pollination, gas chromatography/mass spectrometry, kettle trap flower, pollen transfer efficiency

## Abstract

Elaborated kettle trap flowers to temporarily detain pollinators evolved independently in several angiosperm lineages. Intensive research on species of *Aristolochia* and *Ceropegia* recently illuminated how these specialized trap flowers attract particular pollinators through chemical deception. Morphologically similar trap flowers evolved in *Riocreuxia*; however, no data about floral rewards, pollinators, and chemical ecology were available for this plant group. Here we provide data on pollination ecology and floral chemistry of *R. torulosa*. Specifically, we determined flower visitors and pollinators, assessed pollen transfer efficiency, and analysed floral scent chemistry. *R. torulosa* flowers are myiophilous and predominantly pollinated by Nematocera. Pollinating Diptera included, in order of decreasing abundance, male and female Sciaridae, Ceratopogonidae, Scatopsidae, Chloropidae, and Phoridae. Approximately 16% of pollen removed from flowers was successfully exported to conspecific stigmas. The flowers emitted mainly ubiquitous terpenoids, most abundantly linalool, furanoid (*Z*)-linalool oxide, and (*E*)-β-ocimene—compounds typical of rewarding flowers and fruits. *R. torulosa* can be considered to use generalized food (and possibly also brood-site) deception to lure small nematocerous Diptera into their flowers. These results suggest that *R. torulosa* has a less specific pollination system than previously reported for other kettle trap flowers but is nevertheless specialized at the level of Diptera suborder Nematocera.

## 1. Introduction

The diversification of angiosperm flowers is driven largely by adaptations to pollinators [1,2]. Some plants have evolved extremely complex floral phenotypes specialized in pollination by particular pollinators. Among such specialized flowers are sophisticated kettle-shaped flowers that temporarily trap pollinators. These evolved independently in many different plant lineages, such as Apocynaceae, Aristolochiaceae, and Araceae (see also [3]) and are generally associated strongly with brood-site deception [4,5]. Though similar deceptive strategies, such as food and brood-site deception, tend to be used by such plants, their chemical profiles are generally highly variable due to the vast diversity of different substrates and/or stages of decay mimicked [5]; however, certain key compounds or compound groups can often be identified especially for brood-site deceptive systems [5].

Within the Apocynaceae, trap flowers have evolved independently in two sister subtribes of the tribe Ceropegieae, i.e., in Stapeliinae and Anisotominae [6]. Members of the species-rich subtribe Stapeliinae (>700 spp.) are distributed in Southern and Eastern Africa, Arabia, Madagascar, India, Thailand, and China [7,8]. About 225 species thereof have distinct and highly elaborate kettle-shaped pitfall flowers with various trapping devices, such as slippery surfaces and trapping trichomes; these species are traditionally recognized as *Ceropegia* L. (but see [9]). Within the strictly African subtribe Anisotominae (4 genera, ~30 spp.), species in the genus *Riocreuxia* Decne. have similar though less elaborate kettle trap flowers lacking trapping trichomes and slippery surfaces.

**Figure 1:**
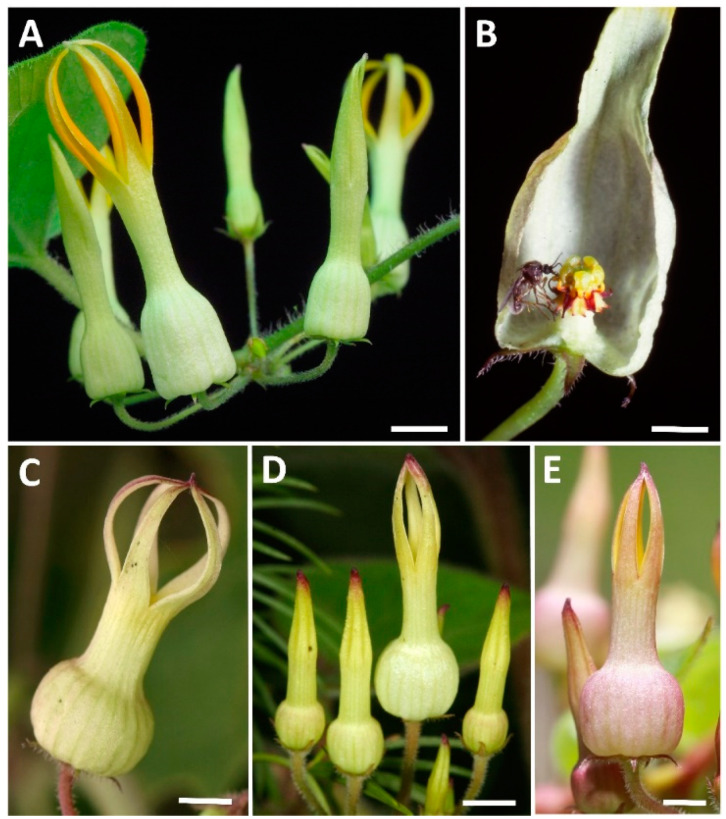
Variability in floral shape and colouration of *Riocreuxia torulosa* plants from different localities in KwaZulu-Natal, South Africa: A, Ashburton, where data for the present study were collected; B, Michel’s Pass at Hogsback, flower opened revealing a ceratopogonid fly caught in the act of removing a pollinarium; C, Ngome; D, Inanda; E, Hermannsburg. Scale bars: 0.5 mm in A and D; 0.3 mm in C and E; 0.2 mm in B. Photographs: Ulrich Meve (**A**), Steven D. Johnson (**B**), and David Styles (**C**–**E**).

*Riocreuxia* was established in 1844 by the French botanist Joseph Decaisne who recognized *R. torulosa* (E. Mey.) Decne., the type species of the genus, as distinct from *Ceropegia*, where it was previously included due to similarities in floral and vegetative characters [10]. *Riocreuxia* is considered to comprise eight species [11]; in some thereof the flowers show extensive intraspecific variation with regard to corolla morphology and the structure of the gynostegium and corona [6,11]. *R. torulosa* (Figure 1) is among the most widespread and most variable species and is considered to be a species complex [6] consisting of two varieties: *R. torulosa* var. *torulosa* and *R. torulosa* var. *bolusii.* As shown by Meve et al. [6], the high levels of morphological variability within *R. torulosa*, and other species of this genus, are not reflected in DNA sequence variation, suggesting rapid recent evolution. In six of the eight *Riocreuxia* species, the corolla is fused to form a kettle trap very similar to that of flowers in *Ceropegia* [12,13]. In *R. aberrans* and *R. chrysochroma*; however, the flowers do not seem to function as a pollinator trap but are rather campanulate with spreading corolla lobe tips [11].

*Riocreuxia* flowers have not been investigated much beyond taxonomical revisions (e.g., [11] and references therein) or phylogenetic analyses (e.g., [6]). By contrast, *Ceropegia* is well studied regarding functional flower morphology [12,14], flower–pollinator relationships [3,15,16,17], and the chemistry behind pollination [18,19,20,21,22]. In *Ceropegia*, the flowers are functionally highly specialized for fly-pollination, and most species only interact with taxa from one or two Diptera families [16,19]. This pollinator specificity is assumed to be achieved by chemical deception [19], and remarkable pollination strategies were identified, such as chemical mimicry of injured honeybees to attract kleptoparasitic flies (e.g., [20]). While pollination has never been studied in *Riocreuxia*, strong morphological similarities to the myiophilous *Ceropegia* trap flowers allow us to assume that *Riocreuxia* flowers are pollinated by Diptera as well. This assumption is supported by the finding of Diptera inside flowers of *R. torulosa* herbarium specimens [11], although these species were not identified and their role as pollinators is unknown.

This study used *R. torulosa* to investigate various aspects of pollination ecology in the genus. The specific objectives of the study were to (1) identify flower-visiting and pollinating insects, (2) determine pollination success in terms of pollen transfer efficiency (PTE), and (3) analyse floral chemistry using dynamic headspace and gas chromatography coupled to mass spectrometry (GC/MS). The data were interpreted in the context of current knowledge of floral chemistry in the closely related genus *Ceropegia*.

## 2. Results

### 2.1. Flower Visitors and Pollinators

The flowers predominantly contained nematocerous Diptera, with ants and small crab spiders (Thomisidae) occasionally found as well. A total of 154 dipteran individuals were collected; they belonged to six different families: Sciaridae (73%), Ceratopogonidae (16%), Scatopsidae (5%), Chloropidae (4%), Phoridae (2%), and Lauxaniidae (1%). The majority (88%) of specimens were females; male flies were represented by 25 individuals (Table 1).

Except for Lauxaniidae, representatives from all families carried pollinaria attached to their mouthparts (Table 1); in total, 43% of the specimens were pollinators. The majority thereof (72%) belonged to the most abundant family Sciaridae. Here, 42% of the specimens (male and female) carried pollinaria; they belonged to an undetermined *Pseudolycoriella* species (32%) and to six *Bradysia* morphospecies (10%) (Table 1). In Ceratopogonidae, 25% of the specimens (9% of all pollinators) carried pollinaria; all these pollinators were females of the same *Forcipomyia* morphospecies (Table 1). In Scatopsidae, 88% of the specimens (11% of all pollinators) carried pollinaria; they were male and female individuals of an undetermined species likely in the genus *Octaseps* and a female individual of an undetermined *Thripomorpha* species (Table 1). In Chloropidae, 33% of the specimens (3% of all pollinators) carried pollinaria; they were females of two morphospecies in undetermined genera (Table 1). In Phoridae, all three specimens (5% of all pollinators) carried pollinaria and were females of an undetermined *Megaselia* species.

### 2.2. Pollination Success

In total, 46% of the investigated flowers (*n* = 501) had pollinaria removed, and 15% were pollinated, i.e., had at least one pollinium inserted. Pollen transfer efficiency (PTE) was 16% with pollinarium removal and pollinium insertion rates of 0.8 ± 0.39 and 0.2 ± 0.13 (mean ± SD/flower/plant), respectively. No nectar was detected in the flowers.

### 2.3. Floral Scent

The average total amount of scent emitted by the flowers was 126 ± 39.8 ng/h, with a minimum of 59 ng/h and a maximum of 144 ng/h (Table 2). The floral scent comprised a total of 24 scent compounds: 10 monoterpenes, 3 sesquiterpenes, 1 irregular terpene, 1 homoterpene, 1 aliphatic compound, 1 aromatic N-containing compound, and 7 unknown compounds (Table 2). The most abundant scent components were the monoterpenoids (*E*)-β-ocimene (31 ± 10.6%) and furanoid (*Z*)-linalool oxide (28 ± 17.5%), an unknown compound (10.8 ± 4.0%), and (*Z*)-3-hexen-1-yl acetate (5.4 ± 8.8%). For several compounds—e.g., (*Z*)-3-hexen-1-yl acetate, (*E*,*E*)-α-farnesene, β-myrcene, linalool, furanoid (*Z*)-linalool oxide—the relative abundances were highly variable among individuals (Table 2).

**Figure 2:**
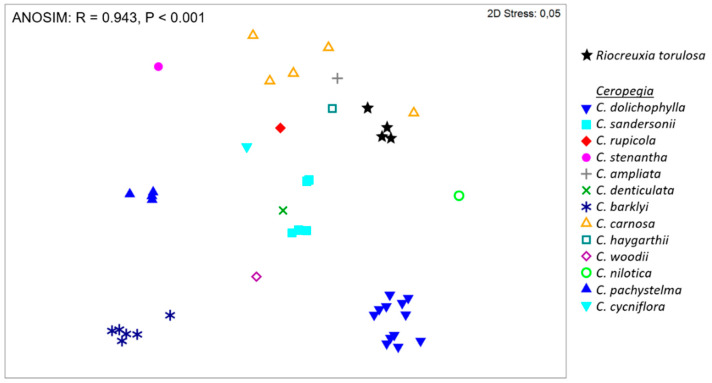
Non-metric multidimensional scaling (NMDS) of scent samples collected from *Riocreuxia torulosa* and different *Ceropegia* species (data taken from [19]) based on semi-quantitative Bray–Curtis similarities.

In pairwise comparisons with *Ceropegia* species, the average dissimilarity in floral scent between *R. torulosa* and any *Ceropegia* species was 98% (ANOSIM: R = 0.943, *p* < 0.001); a total of 10 compounds were also present in the floral scent of *Ceropegia* (see Table 2; see also [19]). In the NMDS analysis, *R. torulosa* grouped in the vicinity of *C. haygarthii C, carnosa,* and *C. ampliata* (Figure 2).

## 3. Discussion

This study documents for the first time that the kettle trap flowers of *Riocreuxia torulosa* are myiophilous. Diptera were the exclusive pollinators, and no other insects/arthropods were observed to visit the flowers. Only occasionally, a flower contained ants or crab spiders, most likely accidentally trapped. The pollinators belonged to a broad spectrum of dipteran taxa in the families Sciaridae, Ceratopogonidae, Chloropidae, Phoridae, and Scatopsidae, with pollen transfer efficiency of 16%. The flowers emitted a floral scent composed of up to 24 compounds the majority of which were widespread floral volatiles known from rewarding plant species.

**Figure 3:**
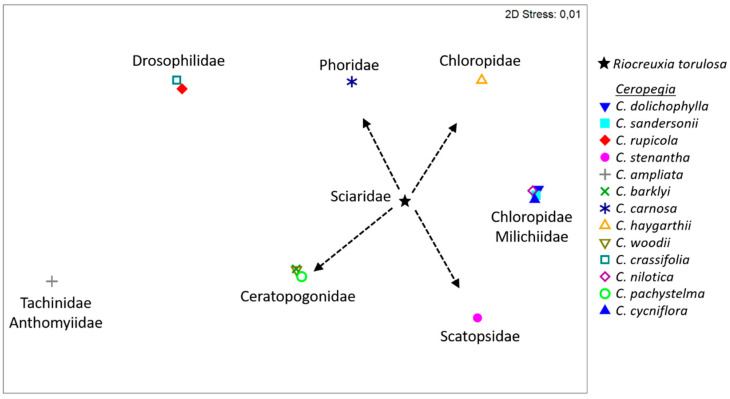
Non-metric multidimensional scaling (NMDS) of pollinator spectra (relative abundancies of Diptera families) of *Ceropegia* species (data taken from [19]) and *Riocreuxia torulosa*. *R. torulosa* was predominantly pollinated by Sciaridae, but also by Ceratopogonidae, Scatopsidae, Chloropidae, and Phoridae. Arrows point towards *Ceropegia* species, which are pollinated by similar Diptera families.

In total, 14 different (morpho)species of five different Diptera families carried pollinaria. All Diptera families and most of the genera to which these species belong are known pollinators of closely related and morphologically similar *Ceropegia* trap flowers (see [16,19]; Figure 3). Ceratopogonidae (*Forcipomyia*) were identified as pollinators of *Ceropegia barklyi*, *C. woodii*, and *C. pachystelma*, and were also commonly trapped inside flowers of *C. haygarthii* [19]. Phoridae (*Megaselia*) were found to pollinate *C. racemosa* and *C. carnosa* [16,19]. Scatopsidae (*Thripomorpha*) were trapped in *C. stenantha* flowers, though did not carry pollinaria. Sciaridae (*Bradysia*) were found inside the flowers of *C. ballyana* and *C. elegans* [16] but did not carry pollinaria, while other Apocynaceae, namely the Brazilian *Ditassa banksii* and *D. burchellii* are pollinated by *Bradysia* species [23,24]. It is not known whether *Ditassa* flowers are deceptive. Representatives of deceptive and pollinator-trapping plant species in other families, such as Aristolochiaceae (*Aristolochia*; [25,26,27]), Araceae (*Arisaema*; [28,29,30,31,32]), and orchids (e.g., *Pleurothallis* [33], *Trichosalpinx* [34], *Lepanthes* [35], *Pterostylis* [36,37]) are likewise known to be visited/pollinated by Phoridae (*Megaselia*), Sciaridae (*Bradysia*, *Corynoptera*, *Pseudolycoriella*), Chloropidae (*Oscinimorpha*), and Ceratopogonidae (*Forcipomyia*). Furthermore, various economically important crops (e.g., *Cacao;* [38]) depend on the same dipteran families and genera for pollination. Though *R. torulosa* shares pollinating dipteran genera with *Ceropegia* (Figure 3) and other plants, the Diptera species might differ among the plants. Comparisons are challenging because many dipteran taxa in the Afrotropics, particularly in super-diverse *Megaselia*, *Forcipomyia, Bradysia*, *Corynoptera*, and *Pseudolycoriella*, are difficult to identify to species level [39,40,41]. The lack of updated taxonomical literature and the vast number of undescribed species hamper proper identification of individuals in these genera [41], and often only morphospecies can be determined, as was the case in this and other studies.

*Riocreuxia torulosa* clearly has a relatively generalized pollination system, as indicated by the number of different Diptera taxa and families found to carry pollinaria. In contrast to *R. torulosa*, most *Ceropegia* trap flowers are extremely specialized and generally exploit only a few species from only 1–2 Diptera families as pollinators [16], though some species are generalized and exploit species from up to 7 families [16]. Pollen transfer efficiency in *R. torulosa* is also high (16%) when compared with both generalized and specialized *Ceropegia* species, where pollen transfer efficiencies are below 10% [17,19,42], with the exception of *C. pachystelma*, which is specialized in pollination by *Forcipomyia* (Ceratopogonidae) and has a noticeably higher pollen transfer efficiency (33%) [19]. It was suggested that the large number of simultaneously open flowers in *C. pachystelma*, as in other plants (e.g., [43,44]), may result in this higher pollination success [19], as could also be the case here in *R. torulosa*, where several flowers, even up to a hundred, are open simultaneously (Figure 1; [11]). Generally, showy mass displays seem to be an important characteristic of generalized food deceptive plants [5], and we assume that the high number of simultaneously open flowers creates a strong chemical signal despite the low amounts of volatiles emitted per single *R. torulosa* flower. This is likely also the case in some *Ceropegia* species with several simultaneously open flowers but low amounts of scent per flower, such as *C. ampliata*, *C. barklyi*, *C. carnosa*, *C. pachystelma*, and *C. woodii*, some of which interestingly share volatiles (e.g., (*E*)-β-ocimene, linalool) with *R. torulosa* [19]. However, a high number of simultaneously open flowers could also translate to many of the insertions being of self-pollinia that would fail to result in seed if the species is self-incompatible.

Members of the Diptera genera trapped in *R. torulosa* flowers are known to use rotting organic material or fungi as oviposition sites or food sources (see [19] and references therein). The flowers of many other plants pollinated by similar Diptera are sapromyiophilous and, to the human nose, emit unpleasant acidic, foul, or mushroom-like odours (see [28,31,45]; own observation for *Ceropegia*), presumably to mimic oviposition sites or food sources for such flies [46,47]. However, the flowers of *R. torulosa* emit a faint sweet-fruity and slightly woody scent, which is not typical for oviposition site mimicry [47], and the chemical profile documented here does not fit a typical sapromyiophilous syndrome [48,49]. It also does not appear to display a sexual deceptive strategy, considering that species of different Diptera families are attracted to the flowers. Therefore, the flowers are interpreted as luring dipteran pollinators primarily through food deception.

Most compounds emitted, including the main compounds linalool, furanoid (*Z*)-linalool oxide, (*E*)-β-ocimene, β-myrcene, limonene, and (*E*,*E*)-α-farnesene, are common, widespread volatiles released from flowers of a variety of different plants [50], and the monoterpene-dominated scent profile would rather suggest attraction of other insects, such as butterflies or bees. Indeed, compounds such as (*E*)-β-ocimene, oxoisophorone, linalool, and linalool-related monoterpenes (furanoid and pyranoid linalool oxide) are known to attract various nectar/pollen feeding insects [51,52,53,54], including Diptera [55,56], to rewarding flowers. It is possible that a wider range of insects are attracted to *R. torulosa* flowers, but that larger insects are filtered out by the narrow entrance to the flower [3,57]. The occasional presence of ants and crab spiders is unlikely volatile-mediated as it was also reported from trap flowers of various *Ceropegia* species, where both ants and crab spiders prey on the pollinators, while ants could also steal nectar [58]. However, *R. torulosa* flowers were not found to secrete any nectar in this study.

We think it most likely that *R. torulosa* flowers deploy a system of generalized food deception, since no nectar or other reward was found to be offered to flower visitors. However, it is also possible that there is a component of brood-site deception as well, because the majority of Diptera were female. It is notoriously difficult to distinguish between food and brood-site deception in plants pollinated by Diptera as they often feed on the same material used for oviposition [5]. Some flowers combine different deceptive strategies to attract pollinators, as *Ceropegia stenantha*, a species exclusively pollinated by scatopsid flies that also emits rather widespread floral volatiles but in combination with unusual compounds, which are believed to be sex pheromones of pollinating Scatopsidae [21]. Most volatiles were found to be electrophysiologically active in both male and female scatopsid pollinators (*Coboldia fuscipes*) [21]. This led to the assumption that generalized food source deception in combination with sexual deception lure male and female Scatopsidae to the flowers of *C. stenantha* [21]. The volatiles emitted by *R. torulosa* differed from those of *C. stenantha* (Figure 2) and 11 of these also elicited consistent antennal responses in male and female *C. fuscipes* flies (Text S1; Figure S1); among these electrophysiologically active compounds were the most abundant and typically reward-signalling compounds: linalool, (*E*)-β-ocimene, and furanoid linalool oxide. Although *C. fuscipes* was not among the Scatopsidae found in *R. torulosa* flowers, these measurements indicated that the tested compounds may be involved in the attraction of Scatopsidae. Similar measurements with species from all pollinating Diptera families found to pollinate *R. torulosa* are necessary to understand the linkage between pollinator spectrum and floral chemistry in this system.

## 4. Conclusions

Our results suggest that *R. torulosa* kettle trap flowers have a deceptive pollination system involving the deployment of generalized reward-indicating compounds to trap both male and female, predominantly nematocerous Diptera, which we assume to be searching for nectar or other sugar-rich plant exudates. However, we cannot exclude the possibility that some of the flies visit flowers in search of brood-sites, as the visitor assemblage was dominated by female insects. Further studies are needed to establish if other species in *Riocreuxia* are also functionally specialized for using Diptera as exclusive pollinators, and if so, which taxa are exploited for pollination and at what level of specialization. Ultimately, comparative studies on pollination and floral chemistry in *Riocreuxia* and other members of the tribe will considerably contribute to our understanding of trap flower evolution in Ceropegieae, especially in combination with phylogenetic data.

## 5. Materials and Methods

### 5.1. Plant Material and Study Sites

*Riocreuxia torulosa* var. *torulosa* is a slender climbing herb [11] that twines up on other vegetation to about 3–4 m in height. The flowers develop in pseudo-umbels with about 5–20 flowers and buds in different developmental stages [11] (Figure 1). During flowering time, many flowers per plant and inflorescence are open simultaneously (Figure 1), and the anthesis of individual flowers lasts about 4 days, with a minimum of 2 days and a maximum of 6 days. As is characteristic for all Asclepiadoideae [7], the flowers are hermaphrodite with male and female reproductive organs being fused to form a gynostegium. Pollen grains are packed in pollinia, two of which are connected via a mechanical clip (corpusculum) to form a pollinarium [7]. The corpusculum must be attached to a pollinator (predominantly insects; see [23]) for pollinarium removal and pollinium deposition (insertion between guide rails; see [12]). In *Riocreuxia*, the gynostegium is situated inside the basal inflation of the kettle trap flower [11] and the pollination mechanism is similar to that of *Ceropegia* kettle trap flowers [12]. *R. torulosa* has a wide distribution range across South Africa. In the present study, a natural population of *R. torulosa* was studied in KwaZulu-Natal (South Africa, SE Pietermaritzburg, Ashburton; voucher—*A. Jürgens, A. Heiduk and U. Meve* 1549; NU, UBT; Figure 1A) from January to May 2013.

### 5.2. Flower Visitors and Pollinators

To obtain information on flower visiting and pollinating insects, flowers were collected from plants in the field and immediately placed in ethanol (99.8%). Trapped insects were removed from these flowers under a dissecting microscope and examined for attached pollinaria. Only those insects with pollinaria attached were denoted as pollinators (see [3]). All collected insects were preserved in ethanol (99.8%) and identified to family, genus, and/or (morpho)species level by taxonomists (F.M., J.-P.H., M.v.T., and John Hash). The examined Diptera specimens were deposited in the collection of the Senckenberg German Entomological Institute (Müncheberg, Germany; Sciaridae) and in the Private Collections of M.v.T. (Bielefeld, Germany; Chloropidae), J.-P.H. (Neuchâtel, Switzerland; Scatopsidae), and A.H. (Salzburg, Austria; other Diptera).

### 5.3. Pollination Success and Natural Seed Set

To assess pollination success, flowers were inspected for pollinaria removal and pollinia insertion. Removal and insertion rates were scored per flower (*n* = 501) and per plant (*n* = 6). From these data, the overall population means of removed pollinaria and inserted pollinia were calculated, and pollen transfer efficiency (PTE) was determined as the percentage of removed pollinia that were inserted between guide-rails (see [59]).

### 5.4. Collection of Floral Volatiles

Floral volatiles were collected in the field using dynamic headspace methods described by Dötterl et al. [60]. Inflorescences with 5–20 open flowers were enclosed in oven bags (polyester; Toppits^®^, Germany; 8 cm × 10 cm) for 30 min. The accumulated floral volatiles (floral headspace) were subsequently collected from the bag for 5 min or 10 min by pulling the air through an adsorbent trap using a membrane pump (G12/01 EB, Rietschle Thomas Inc., Puchheim, Germany) with a flow rate set to 200 mL/min). The adsorbent traps were made of quartz microvials (ChromatoProbe; Varian Inc., Palo Alto, CA, USA; 15 mm length; 2mm inner diameter; closed end cut open) filled with a mixture (1:1, *v*/*v*) of Tenax-TA (mesh 60–80) and Carbotrap B (mesh 20–40) (Supelco, Bellefonte, PA, USA). The microvials were plugged with glass wool from both ends to keep the Tenax-Carbotrap mixture in place. In addition, samples of ambient air were collected in order to distinguish between floral volatiles and those in surrounding air (see [22]).

### 5.5. Chemical Analyses

The floral headspace (HS) samples of *R. torulosa* were analysed by gas chromatography/mass spectrometry (GC/MS). A Bruker 450 GC (Varian, Palo Alto, CA, USA) was fitted with a 30 m DB5 column (Bruker; 0.25 mm inner diameter; 0.25 µm film thickness) and coupled via an 11 m DB1 column (Bruker; film thickness, 0.25 µm) to a Bruker 350 quadrupole MS. Mass spectra were obtained at 70 eV in electron ionization mode. The microvials containing the HS volatiles were introduced into a Varian 1079 injector (Varian Inc., Palo Alto, CA, USA) fitted with a thermal desorption device (Chromatoprobe) [61]. The carrier gas (helium) was set to a flow of 1.6 mL/min. The injector was first kept at 50 °C for 2 min (20:1 split); for thermal desorption, the temperature was then increased to 200 °C (200 °C/min; splitless mode). The GC oven temperature, after an initial hold at 50 °C for 3 min, was ramped up to 240 °C (10 °C/min) and held at this temperature for 12 min.

Scent components were identified using several mass spectral data bases: FFNSC 2, MassFinder 3, NIST 11, Wiley 9, and Adams [62]. Published Kovats retention indices (KRI) and, whenever possible, retention times and mass spectra of authentic standards were used to verify the identity of the scent components. For quantification of compounds, specific amounts of synthetic standards (applied to small adsorbent tubes) were injected and the mean response (peak area) of these compounds was used to estimate the amounts of floral volatiles in the samples (see [60]).

### 5.6. Statistical Analyses

The flower scent of *R. torulosa* was tested for (dis)similarities with the floral scent of *Ceropegia* species (data taken from [19]). The Bray–Curtis (BC) similarity index was calculated with the relative amounts of floral volatiles per species and sample, using Primer 6.1.11 [63]. Based on the BC-matrix, an ANOSIM (Factor: Species; 10,000 permutations) was performed using the same software package to test for differences in scent among and within species. The variation among samples was visualized using non-metric multidimensional scaling (NMDS) in Primer. In addition, one-way SIMPER was used in Primer to determine the compounds responsible for the semi-quantitative differences in scent that were found among species. Furthermore, NMDS was used to visualize (dis)similarities in pollinating Diptera families between *Ceropegia* species (data taken from [19]) and *R. torulosa.*

## Figures and Tables

**Table 1:** Flower visiting and pollinating Diptera collected from kettle trap flowers of *Riocreuxia torulosa*. In brackets: number of Diptera with pollinaria attached to mouthparts. Pollinating taxa are printed in bold.

Total Number of Diptera:	Male	Female
	24 (12)	130 (52)
CERATOPOGONIDAE	2	22 (6)
***Forcipomyia*** Meigen, 1818 **sp. 1**		15 (6)
*Forcipomyia* sp. 2		1
*Forcipomyia* sp. 3		2
Undetermined sp. 1	1	
Undetermined sp. 2		1
Undetermined sp. 3	1	3
CHLOROPIDAE	1	5 (2)
*Oscinimorpha* cf. *minutissima* (Strobl, 1900)	1	
Undetermined sp. 1		3
**Undetermined sp. 2**		1 (1)
**Undetermined sp. 3**		1 (1)
LAUXANIIDAE		1
*Sapromyza* Fallén, 1810 sp.		1
PHORIDAE		3 (3)
***Megaselia*** Rondani, 1856 **sp.**		3(3)
SCATOPSIDAE	3 (2)	5 (5)
***Octaseps*** Haenni and Amorim, 2016 **sp. nov.** aff. *labellata* Cook, 1965	3 (2)	4 (4)
***Thripomorpha*** Enderlein, 1905 sp.		1 (1)
SCIARIDAE	18 (10)	94 (37)
*Bradysia* Winnertz, 1867 sp. 1 (*Bradysia fallaciosa* group)	3	1
*Bradysia* sp. 2 (*Bradysia fallaciosa* group)		1
***Bradysia*****sp. 3** (*Bradysia hilaris* group)	1 (1)	6 (3)
*Bradysia* sp. 4		11
***Bradysia*** **sp. 5**		3 (2)
***Bradysia*** **sp. 6**		3 (2)
***Bradysia*** **sp. 7**		1 (1)
*Bradysia* sp. 8		1
*Bradysia* sp. 9		1
***Bradysia*** **sp. 10**		1 (1)
***Bradysia*** **sp. 11**		1 (1)
*Corynoptera* Winnertz, 1867 sp.		10
***Pseudolycoriella*** Menzel & Mohrig, 1998 **sp.**	14 (9)	54 (27)

**Table 2:** Floral volatiles (relative amounts) identified in samples collected from *Riocreuxia torulosa*. Four samples were taken from four different plants (A–D); the number of flowers per sample, total sampling time, and total amount of scent per flower are indicated. The occurrence of the compounds in closely related *Ceropegia* species (see [19]) is also shown. Values in bold, >5%; tr, <0.05%; san, *C. sandersonii*; rup, *C. rupicola*; amp, *C. ampliata*; den, *C. denticulata*; car, *C. carnosa*; hay, *C. haygarthii*; cyc, *C. cycniflora*; nil, *C. nilotica*.

Chemical Compound	A (7 _flowers_)	B (6 _flowers_)	C (5 _flowers_)	D (20 _flowers_)	*Ceropegia* Species with Similar Floral Compounds
Total sampling time [min]:	35	40	35	35	
Total scent emitted per flower [ng/h]:	58.9	143.7	140.9	161.9	
***Aliphatics***					
(*Z*)-3-Hexen-1-yl acetate ^S,EAD^	0.2	0.3	**20.5**	0.4	san, rup, amp, den, car, hay, cyc
***Aromatics***					
Phenylacetonitrile	1.4	1.4	1.1	1.8	den
***Terpenoids***					
***Monoterpenes***					
Limonene ^S,EAD^	1	0.9	0.6	0.5	nil
Linalool ^S,EAD^	**9.6**	**5.1**	2.9	**7.5**	san, rup, amp, hay
Furanoid (*E*)-linalool oxide ^S,EAD^	2.5	3.6	0.8	2.8	
Furanoid (*Z*)-linalool oxide ^S,EAD^	**35.9**	**5.6**	**19.5**	**52.4**	
Pyranoid (*E*) + (*Z*)-linalool oxide ^S,EAD^	2.8	3.6	1.6	3.5	
β-Myrcene ^S,EAD^	0.5	**7.9**	2.9	**5.2**	den
Myrcenol	0	0	0	tr	
(*E*)-β-Ocimene ^S,EAD^	**34.6**	**38.6**	**39.2**	**13.3**	san, amp, den, car, hay
(*Z*)-β-Ocimene	0.8	0.7	0.3	0.5	san
***Sesquiterpenes***					
α-Farnesene (isomer not assigned)	0.4	1.7	0.1	0.1	
(*E*,*E*)-α-Farnesene	1.4	**10.9**	0.7	1	san
(*E*)-β-Farnesene ^S,EAD^	0.1	0.2	tr	tr	
***Irregular terpenes***					
4-Oxoisophorone ^S,EAD^	0.5	0.3	0.6	0.5	den
***Homoterpenes***					
(*E*)-4,8-Dimethyl-1,3,7-nonatriene ^S^	0.6	0.3	1.5	0.1	san, amp, car, hay
***Unknowns*** ^(**7 in total**)^					
m/z: 43, 71, 41, 55/59	**8**	**17.5**	**7.4**	**10.3**	
Minor unknowns ^a^	**0.12**	**1.36**	**0.24**	**0.36**	

^a^ number of unknown compounds pooled per sample; ^S^ compound verified with authentic standard; ^EAD^ compound electrophysiologically active (see Supplementary Materials: Text S1, Figure S1).

## Supplementary Materials

The following are available online at https://www.mdpi.com/article/10.3390/plants10081564/s1: Text S1: Methods and Results for electrophysiological measurements, Figure S1: Antennal responses of a female and a male *Coboldia fuscipes* (Scatopsidae) fly to synthetic scent compounds of *Riocreuxia torulosa*.
