# Supplementary material for: Fly Pollination of Kettle Trap Flowers of Riocreuxia torulosa (Ceropegieae-Anisotominae): A Generalized System of Floral Deception
Source: Plants (Basel). 2021 Jul 29;10(8):1564. doi: 10.3390/plants10081564 (PMC8398993; doi:10.3390/plants10081564)
Supplement: Supplementary file 1 [file plants-10-01564-s001.zip › Text S1.pdf]

Supplemental Material

Electrophysiological measurements – GC/EAD

We performed electrophysiological measurements with antenna of *Coboldia fuscipes* (Scatopsidae) flies which were bred in Salzburg (Austria). *C. fuscipes* was not among the Scatopsidae found inside the flowers of *R. torulosa* in the plant's natural range in South Africa. However, as exemplified with studies on *Ceropegia stenantha* [1] measurements with non-natural pollinators can be an important function of the tested volatiles in attraction of natural pollinators.

Electrophysiological measurements were performed with an Agilent 7890A gas chromatograph (Santa Clara, California, USA) equipped with a Zebron ZB-5 analytical column (5% phenyl polysiloxane; length: 30 m, inner diameter: 0.32 mm, film thickness: 0.25 µm, Phenomenex), a flame ionization detector (FID), and a setup for electroantennographic detection (EAD) (for details see [1]). Antenna of *C. fuscipes* flies were tested to eleven components identified in the floral scent samples of *Riocreuxia torulosa* (see Results): ß-myrcene, (*Z*)-3-hexenyl acetate, limonene, (*E*)-β-ocimene, (*Z*)- and (*E*)-linalool oxide furanoid, (*Z*)- and (*E*)-linalool oxide pyranoid, linalool, 4-oxoisophorone, and (*E*)-ß-farnesene. The compounds were tested as a mixture of synthetic standards (Sigma Aldrich; purity: 90–99%), diluted in acetone (SupraSolv, Merck KgaA, Germany; equal volumes).

Antenna of seven male and five female scatopsid individuals were tested in 12 runs (one antenna per individual). In each run 1 µl (10^{-3}) of the sample was injected in splitless mode (injector temperature: 250°C; oven temperature: 40°C). After 30 seconds the split opened and the oven gradually (10°C/min) heated up to 220°C. A µFlow splitter (Gerstel, Mühlheim, Germany; nitrogen was used as make-up gas) connected the column to two deactivated capillaries of which one (length: 2 m, inner diameter: 0.15 mm) lead to the FID setup, and the other one (length: 1.0 m, inner diameter: 0.2 mm) to the EAD setup. The EAD outlet was placed in a cleaned, humidified air flow directed over the antenna.

Each scatopsid fly used for measurements was anaesthetized (CO_2) before its head was cut off under a dissecting microscope. Two glass micropipettes were filled with insect Ringer's solution (8.0 g/l NaCl, 0.4 g/l KCl, 4.0 g/l $CaCl_2$), connected to silver wires, and placed in stainless steel electrode holders. The reference electrode was connected to the caudal side of the flies' head and the recording electrode was in contact with the antenna tip. Because the antennae were densely covered with microtrichia, the last flagellomere was cut at the very tip for better immersion of the antennae into Ringer's solution. The antenna was placed in the stream of filtered, humidified air carrying the volatiles from the EAD outlet. Antennal responses were recorded with a two-channel USB intelligent data acquisition controller (IDAC-2). To identify the electrophysiologically active compounds, 1.0 µl of the synthetic mixture was applied to an adsorbent tube and analyzed by GC/MS (see above).

Results of electrophysiological measurements

Both female and male *Coboldia fuscipes* flies consistently responded to all eleven tested synthetic scent components of *Riocreuxia torulosa* (Figure S1, Table 2). The strongest antennal responses were elicited by (*Z*)-3-hexenyl acetate, (*Z*)- and (*E*)-linalool oxide furanoid, linalool, 4-oxoisophorone, (*Z*)-linalool oxide puranoid, and (*E*)-β-farnesene.

References

1. Heiduk, A.; Haenni, J.-P.; Meve, U.; Schulz, S.; Dötterl, S. Flower scent of *Ceropegia stenantha*: electrophysiological activity and synthesis of novel components. *J. Comp. Physiol. A* **2019**, *205*, 301–310, doi:10.1007/s00359-019-01318-4.
