# Supplementary figures and images for: Fly Pollination of Kettle Trap Flowers of Riocreuxia torulosa (Ceropegieae-Anisotominae): A Generalized System of Floral Deception
Source: Plants (Basel). 2021 Jul 29;10(8):1564. doi: 10.3390/plants10081564 (PMC8398993; doi:10.3390/plants10081564)

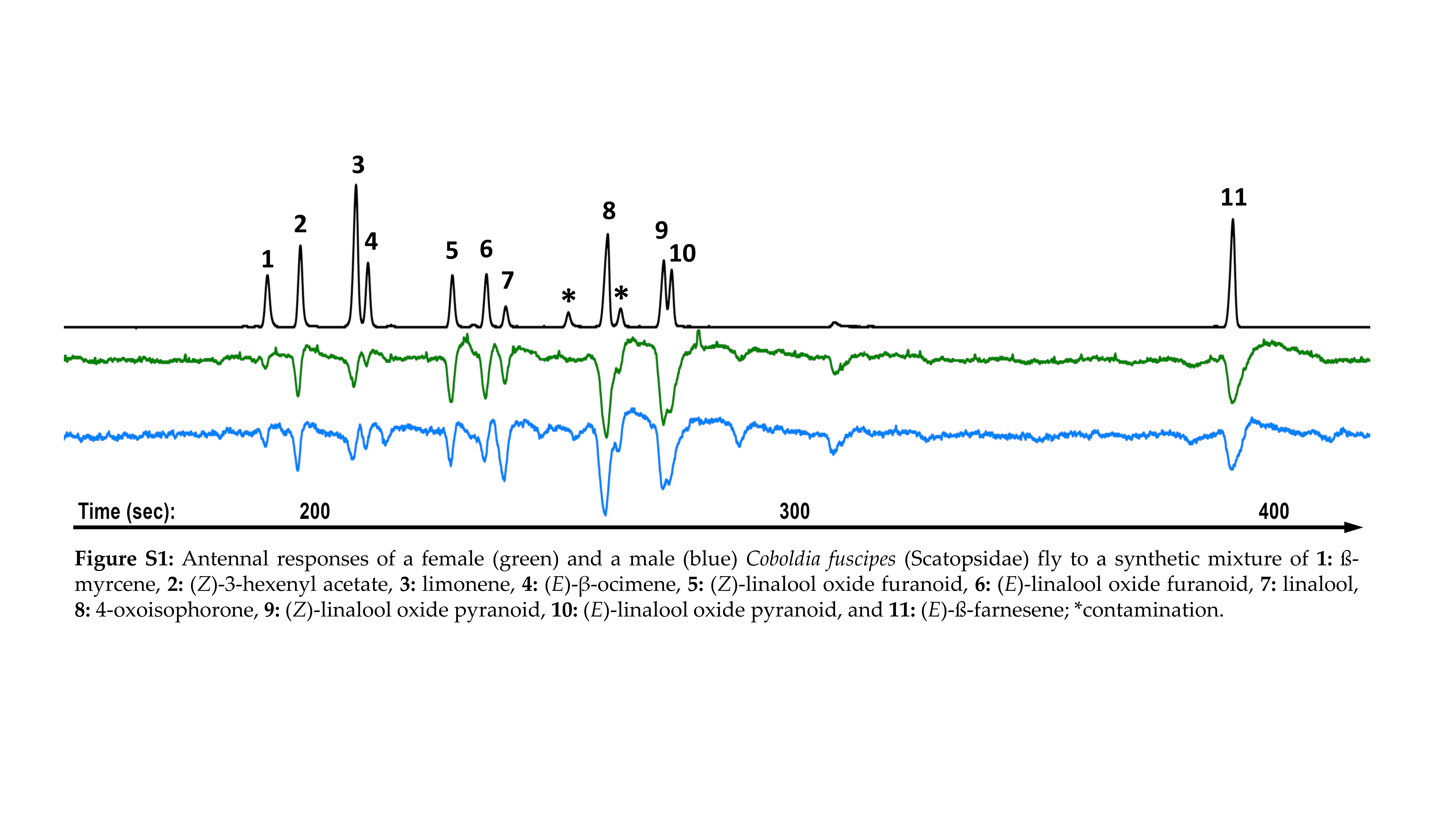

Supplement: Supplementary file 1 [file plants-10-01564-s001.zip › FigureS1_EAD-Riocreuxia.tif]
